# Catatonia and Delirium: Similarity and Overlap of Acute Brain Dysfunction

**DOI:** 10.3389/fpsyt.2022.876727

**Published:** 2022-04-07

**Authors:** Masako Tachibana, Kanako Ishizuka, Toshiya Inada

**Affiliations:** ^1^Department of Psychiatry, Nagoya University Hospital, Nagoya, Japan; ^2^Health Support Center, Nagoya Institute of Technology, Nagoya, Japan; ^3^Department of Psychiatry, Nagoya University Graduate School of Medicine, Nagoya, Japan

**Keywords:** catatonia, delirium, acute brain dysfunction, diagnostic concepts, overlap

Catatonia and delirium were originally different diagnostic concepts, but in recent years, their similarities have been highlighted. Catatonia was first described by Kahlbaum in 1874 as a psychosis with specific physical tension (1) and regarded as an important disease type, mainly within the framework of schizophrenia (2). However, catatonia is seen in not only schizophrenia but also neurodevelopmental, bipolar, and depressive disorders, as well as other medical conditions, and it has come to be regarded as a catatonic syndrome (3, 4). The Diagnostic and Statistical Manual of Mental Disorders, Fifth Edition (DSM-5) has developed common diagnostic criteria for catatonia due to several disorders, differentiating between catatonia due to a mental disorder and another medical condition (5). Recently, catatonia due to a general medical condition has been more frequent than due to a mental disorder (6).

Delirium was described by Lipowski in 1987 as acute global brain dysfunction occurring in the setting of physical illness (7). Delirium is defined in the DSM-5 as a disturbance in attention and awareness (reduced orientation to the environment) (5) and is thought to be caused by physical factors or drugs (8).

The origins of the two diagnostic concepts of catatonia and delirium are different, and the DSM-5 description of catatonia due to a general medical condition specifies that “the disturbance does not occur exclusively during the course of a delirium” (5). However, as the diagnostic concept of catatonia has shifted, the similarities between the symptoms of catatonia and delirium have been noted. Jaimes-Albornoz and Serra-Mestres (9) studied catatonic phenomena in 112 patients aged over 65 years who were referred to a psychiatry service in a general hospital. Seven patients (6.3%) met the DSM-IV criteria for catatonia, and three of these patients presented concomitant delirium. Grover et al. (10) studied the prevalence of catatonic symptoms in 205 delirium patients. Symptoms common to catatonia were found, including excitement (72.7%), immobility or stupor (21.4%), mutism (15.6%), and negativism (10%). Of the delirium patients, 12.7% met the DSM-5 criteria for catatonia. Connell et al. (11) studied the occurrence of catatonia and delirium in 378 critically ill patients. Of these patients, 88 (23%) met the diagnostic criteria for catatonia, and 250 (66%) met the criteria for delirium, using the DSM-5. Of the 88 2 patients who experienced catatonia, 82 (93.2%) simultaneously experienced delirium. In clinical practice, many cases are difficult to differentiate (12, 13), and the current clinical issue is how to consider the overlap of symptoms between catatonia and delirium. The specificity of the diagnostic criteria of the DSM-5 has been criticized (6, 14). The International Classification of Diseases, 10th Revision (ICD-10) describes organic catatonic disorder: “It has not been conclusively determined whether an organic catatonic state may occur in clear consciousness or whether it is always a manifestation of delirium, with subsequent partial or total amnesia” (15). The concept of a catatonia–delirium spectrum has even been suggested (4).

Differentiating between catatonia and delirium is necessary because they have different treatment strategies. Catatonia is responsive to certain treatments such as benzodiazepines and modified electroconvulsive therapy (16), which are effective yet unspecific in their modes of action (17). However, both may also produce or exacerbate delirium, and conversely, antipsychotics used to treat symptoms of delirium may produce or exacerbate catatonia (13). Catatonia due to a mental disorder is not accompanied by disturbance of consciousness; memory is thought to be maintained even when symptoms appear in many instances. However, delirium is a type of disturbance of consciousness, and memory is thought to be impaired. Some patients recall their experiences after recovering from delirium (18, 19), and in catatonia due to a medical condition, consciousness is often impaired due to the influence of the medical condition, and some catatonia due to mental disorder, such as malignant catatonia, can display altered consciousness with autonomic instability (20). Electroencephalography (EEG) remains the most widely available physiological measure of delirium and reveals diffuse background slowing, typically in the delta range (8). Most catatonia tends to present with a normal EEG, but in one study, over 80% of catatonia caused by drugs or physical illness exhibited abnormal EEG findings, the most common being diffuse slowing (4). Two thirds of medical catatonia involved central nervous system (CNS)-specific disease, including encephalitis, neural injury, developmental disorders, structural brain pathology, or seizures. Whenever medical catatonia is considered, a workup directed at CNS-specific pathology is necessary (21). In addition, the presence of delirium strongly predicts a medical cause of catatonic features (4, 21), and a comprehensive evaluation for potential medical causes should be performed in suspected cases of both delirium and catatonia (Figure 1). Even in catatonia patients with psychiatric disorders, recent neuroimaging findings indicate increased neuronal activity in premotor areas, reduced GABA-A receptor density, and poor connectivity within the motor circuit (17). Thus, acute brain dysfunction includes both delirium and non-functional catatonia, and they often overlap. Further research is needed to better elucidate the specific contributions of the various potential mechanisms associated with both. It should also prompt a reevaluation of the conceptual model of acute brain dysfunction.

**Figure 1:**
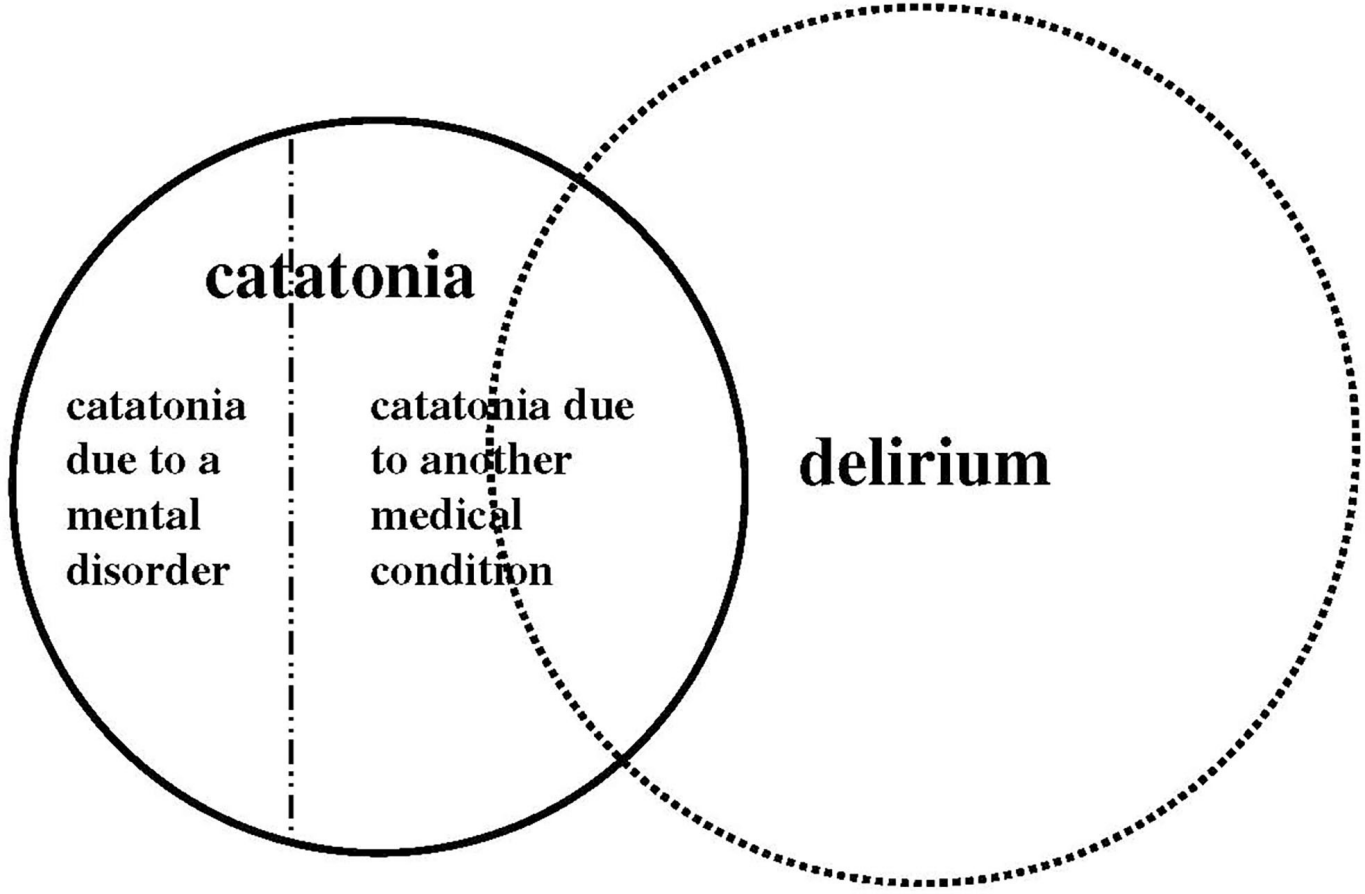
Schematic image of the overlap of symptoms between catatonia and delirium according to the DSM-5 diagnostic criteria.

## Author Contributions

MT, KI, and TI planned, organized, and selected the papers be included in the special issue. All authors contributed to the article and approved the submitted version.

## Funding

This work was partly supported by JSPS KAKENHI Grant Number JP19K08071.

## Conflict of Interest

The authors declare that the research was conducted in the absence of any commercial or financial relationships that could be construed as a potential conflict of interest.

## Publisher's Note

All claims expressed in this article are solely those of the authors and do not necessarily represent those of their affiliated organizations, or those of the publisher, the editors and the reviewers. Any product that may be evaluated in this article, or claim that may be made by its manufacturer, is not guaranteed or endorsed by the publisher.
